# High intensity signal on MIP images from routine TOF-MRA of carotid atherosclerotic plaque indicates higher volume of intraplaque hemorrhage and lipid rich necrotic core

**DOI:** 10.1186/1532-429X-14-S1-P133

**Published:** 2012-02-01

**Authors:** Kiyofumi Yamada, Yan Song, Jie Sun, Li Dong, Dongxiang Xu, Daniei S Hippe, Marina S Ferguson, Baocheng Chu, Thomas S Hatsukami, Min Chen, Cheng Zhou, Chun Yuan

**Affiliations:** 1 Radiology, University of Washington, Seattle, WA, USA; 2 Radiology, Beijing Hospital, Beijing, China; 3 Surgery, University of Washington, Seattle, WA, USA

## Background

Carotid intraplaque haemorrhage (IPH) and lipid rich necrotic core (LRNC) have been associated with accelerated plaque growth, luminal narrowing, future surface disruption and development of symptomatic events. It has also been reported that unstable plaque which contains LRNC or IPH is associated with an increased number of emboli after carotid artery stenting. Therefore, a simple screening method to detect these components in the plaque is needed. Maximum intensity projection (MIP) images from time-of-flight MR angiography (TOF-MRA) are widely and routinely used for screening carotid artery stenosis. This study examined whether high-intensity signal (HIS) in the plaque on MIP images from routine TOF-MRA could quantify IPH.

## Methods

Seventy six patients with a diagnosis of carotid artery stenosis underwent carotid MR imaging. Two experienced reviewers first assessed the presence of HIS in the plaque on MIP images from TOF-MRA and then, blinded to the results, assessed plaque component volumes (IPH, LRNC, Fibrous tissue and Calcification volume) on multicontrast cross sectional MRI using a specialized software suite for plaque analysis.

## Results

Eight carotid plaques produced HIS in the plaque on MIP images from TOF-MRA. In the HIS-positive plaque group (P group; n=8), IPH volume and LRNC volume were significantly higher than those in the HIS negative group (N group; n=68) (IPH; 142.8±97.7mm3 vs 13.4±36.1 mm3, P<0.001. LRNC; 379.8±203.4 mm3 vs 106.4±122.1 mm3, p<0.001). There were no differences in the fibrous tissue and calcification volume between the P and N groups.

## Conclusions

Our results strongly suggest an association between the presence of carotid LRNC with IPH and HIS on MIP images from TOF-MRA. TOF-MRA is routinely used in atherosclerosis screening and the validation of high signal on MIP images has created a valuable tool in the assessment of carotid plaque vulnerability.

## Funding

None.

**Figure 1:**
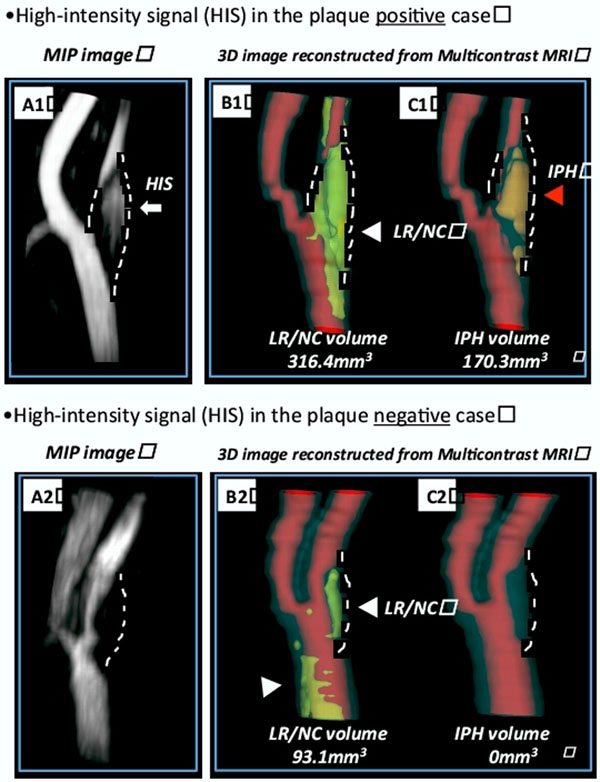
Representative images of high-intensity signal (HIS) positive plaque (A1) and negative plaque (A2). HIS positive plaque is identified as high signal in the vessel wall without connection to the lumen (White arrow). HIS negative plaque has no HIS but shows luminal stenosis. Lipid rich necrotic core (LRNC) (B1&2; ehite arrowhead) and intraplaque haemorrhage (IPH) (C1; Red arrowhead) were displayed using three dimensional reconstruction images of multicontrast MRI. Volume of LRNC and IPH are calculated from cross-sectional areas length of plaque using the computer-aided system for cardiovascular disease evaluation (CASCADE), a specialized software suite for plaque analysis.

